# Robotic Thymectomy for Myasthenia Gravis: Analysis of the Surgical and Neurological Outcomes After a 20 Years' Experience

**DOI:** 10.1111/ene.70147

**Published:** 2025-04-15

**Authors:** Giovanni M. Comacchio, Marco Schiavon, Luca Bello, Marco Mammana, Eleonora Faccioli, Elena Pegoraro, Giulia Lorenzoni, Giorgio Cannone, Giuseppe Cataldi, Giulia Pagliarini, Alessandro Rebusso, Samuele Nicotra, Dario Gregori, Maria Carlotta Marino, Giuliana Capece, Pietro Riguzzi, Federica Pezzuto, Fiorella Calabrese, Andrea Dell'Amore, Federico Rea

**Affiliations:** ^1^ Thoracic Surgery Unit University Hospital of Padua Padua Italy; ^2^ Department of Neurosciences DNS University of Padua Padua Italy; ^3^ Unit of Biostatistics, Epidemiology and Public Health, Department of Cardiac, Thoracic, Vascular Sciences and Public Health University of Padua Padua Italy; ^4^ Pathology Unit, Department of Cardiac, Thoracic, Vascular Sciences and Public Health University of Padua Padua Italy

**Keywords:** myasthenia gravis, remission, surgery

## Abstract

**Background:**

Evidence supporting robotic thymectomy for myasthenia gravis is generally based on small sample‐size studies, heterogeneous in patient selection and in reporting outcomes. Therefore, this study was conducted to assess the surgical and neurological outcomes of robotic thymectomy in myasthenic patients and to identify prognostic factors associated with symptoms' remission through a large cohort of patients operated in a 20 years' period.

**Methods:**

A retrospective analysis of a prospectively maintained database was conducted for all patients undergoing robotic thymectomy for myasthenia gravis between 2002 and 2022. Myasthenia Gravis Foundation of America (MGFA) recommendations were used to report the neurological outcomes. Complete remission and overall improvement were evaluated using Cumulative Incidence Functions, while the effect of preoperative variables on the probability of remission was estimated with Cox models.

**Results:**

In total, 267 patients underwent robotic thymectomy. Median operative time was 135 min and there were 7 (2.6%) open conversions. Clinical follow‐up (median 83 months) showed a 5‐year probability of complete remission of 18% and of overall improvement of 84%. Complete remission was negatively associated with age (HR 0.97, 95% CI 0.95–0.99, *p* = 0.001) and preoperative use of pyridostigmine (HR 0.34, 95% CI 0.15–0.81, *p* = 0.014), while severe MGFA class did not reach significance (HR 0.55, 95% CI 0.3–1.01, *p* = 0.052). Instead, there was a benefit in patients operated on in later years (HR 1.11, 95% CI 1.04–1.18, *p* = 0.01).

**Comment:**

Robotic thymectomy is a safe procedure. Long‐term neurological follow‐up demonstrated an improvement in most patients, also in subgroups that historically showed worse outcomes.

## 1 Introduction

Myasthenia gravis (MG) is a rare acquired autoimmune disease affecting the neuromuscular junction and causing variable degrees of fatigable weakness of skeletal muscles [1]. The treatment of MG has greatly evolved over time, including symptomatic treatments (anticholinesterase inhibitors), immunosuppressants, immunomodulators, monoclonal antibodies, and other biological treatments, and the surgical treatment with thymectomy [2]. Different retrospective studies have described thymic resection as an effective treatment for MG, but it was only in 2016 that the results of the first prospective randomized trial (MGTX) were published and demonstrated the benefits of thymectomy compared with medical therapy alone [3, 4]. However, this study considered only patients positive for antibodies against acetylcholine receptor (AbAchR), excluded those with pure ocular MG, and allowed drawing conclusions only for patients undergoing transsternal thymectomy [4]. On the contrary, in recent years minimally invasive thymectomy (thoracoscopic, robotic) gained attention because of the optimal surgical outcomes compared with open transsternal thymectomy, together with apparently equal neurological outcomes [5]. However, these reports are frequently characterized by small sample sizes, different definitions of remission and extent of resection, and short follow‐up [6, 7, 8, 9, 10, 11, 12]. Therefore, in the absence of a randomized trial including these new approaches, the role of minimally invasive thymectomy still remains controversial. Needing larger and more consistent data, our present study was specifically conducted to evaluate the surgical and long‐term outcomes of robotic thymectomy, analyzing our 20 years' experience.

## 2 Patients and Methods

We performed a retrospective review of a prospectively maintained database for all patients undergoing robotic thymectomy and with a diagnosis of MG at the Thoracic Surgery Unit of the University Hospital of Padua, Italy, between April 2002 and April 2022. Robotic thymectomy represents our standard technique in all patients with MG, except for those with large size or invasive/advanced thymic tumors, that were therefore excluded from the study. This study was approved by the Institutional Review Board (449n/AO/23, 18/01/2023) and all patients gave informed consent. Additionally, the study was conducted according to the Declaration of Helsinki.

Information on patient demographics, preoperative and postoperative medical therapy for MG, surgical intra‐and postoperative data (e.g., complications, open conversion, operative time, hospital stay), neurological and oncological data were collected.

All patients were evaluated by a neurologist and the diagnosis of MG was made according to the presence of symptoms, autoantibody status, electrophysiological tests, or response to therapy. Patients that tested negative for AbAchR were tested for anti‐MuSK antibodies and excluded from surgery when positive. Patients with symptoms affecting daily activities or quality of life were prescribed pyridostigmine, up to a dose of 60 mg 3–4 times daily. Patients with significant symptoms upon waking up in the morning were advised to take slow‐release pyridostigmine at bedtime, starting with 90 mg and eventually increasing to 180 mg. Dose titration was based on clinical response and presence of side effects. Before surgery, patients were required to be in a stable control of symptoms; plasma exchange therapy and intravenous immunoglobulin treatment were administered in the preoperative period to patients at higher risk for postoperative myasthenic crisis based on the neurologist's judgment.

The Myasthenia Gravis Foundation of America (MGFA) recommendations were used to stratify the preoperative class of MG [13]. Thymic tumors were staged according to the Masaoka‐Koga system, while the World Health Organization classification was used for histological definition [14, 15].

Patients were followed up both by neurologists and surgeons and, if they stopped visiting our center, were called for follow‐up. Those who reached stable remissions were advised to gradually taper corticosteroid and/or other treatments, and followed up every 6 months or earlier in the case of symptom relapse. Symptomatic patients were given tailored treatment and followed up after a time deemed appropriate by the neurologist. Based on each patient's neurologist evaluation, on the response to a questionnaire, and on current medications, the therapeutic effect was assessed according to the MGFA Post‐Intervention Status (PIS) [13]. Also, the change in status was assessed according to MGFA‐PIS, with improvement accounting for patients with complete stable remission (CSR), pharmacologic remission (PR) and minimal manifestations (MM). These endpoints were calculated at the end of a minimal 12‐month follow‐up period.

### 2.1 Surgical Technique

We prefer a left‐sided three‐port technique for robotic thymectomy in all patients with non‐thymomatous MG using the “daVinci” robotic system (Intuitive Surgical Inc., Sunnyvalley, CA). For patients with thymoma, the side of approach is chosen according to the location of the lesion. The surgical technique has been already described [6, 16]. Briefly, we perform an en‐bloc resection of the thymic gland, the thymic tumor (if any), and all the mediastinal adipose tissue between the diaphragm caudally, the thyroid gland cranially, and the phrenic nerves laterally (extended thymectomy). Particular attention is paid to the aortocaval groove and the aortopulmonary window. We cannot ensure a complete resection only of the cardiophrenic fat pad on the side opposite to the access, as for all lateral approaches. The no‐touch technique is always applied in case of neoplastic lesions.

### 2.2 Statistical Analysis

Descriptive statistics were reported as median (I‐III interquartile range, IQR) for continuous variables and as absolute numbers (percentages) for categorical variables.

Cumulative incidence of CSR and of overall improvement at follow‐up was evaluated using Cumulative Incidence Functions (CIFs) to account for competing risks. The association of preoperative variables with the outcomes of interest was evaluated using univariable and multivariable Cox proportional hazards models, presenting the results as Hazard Ratio (HR), 95% confidence interval (95% CI), and *p*‐value. To assess the changes in the dosage of steroids and pyridostigmine administered at follow‐up, and the variation over time of the specimens' weight, univariable Gamma models were estimated to account for non‐normal outcome distribution. The marginal effect was computed considering the partial derivatives of the marginal expectation. Results were reported as the average marginal effect (AME), 95% CI, and *p*‐value. The statistical significance was set at *p* < 0.05. The analyses were carried out with the R software program (version 4.3.1).

## 3 Results

During the study period, 267 patients underwent robotic thymectomy for MG. Figure S1 shows the number of patients operated on each year of activity. There were 81 (30%) male and 186 (70%) female patients, with a median age of 42 years. Distribution of preoperative MGFA class is reported in Table 1. Median duration of symptoms before surgery was 11 months, and 198 (74%) of patients tested positive for AbAchR. Seronegative patients were younger than seropositive ones (*p* = 0.003), had comparable MGFA class distribution (*p* = 0.1), but were more frequently treated with cyclosporine (*p* = 0.015) (Table S1).

**TABLE 1:** Clinical characteristics of the patients.

Variable	Study population (*n* = 267)
Sex, *n* (%)	
F	186 (70%)
M	81 (30%)
Age at surgery, years, median (IQR)	42 (30–53)
Preoperative MGFA, *n* (%)	
I	57 (23%)
II	113 (46%)
III	59 (24%)
IV	18 (7%)
Symptoms duration, months, median (IQR)	11 (6–20)
Antibody anti‐AchR status, *n* (%)	
Positive	198 (74%)
Seronegative	63 (24%)
Unknown	6 (2%)
Preoperative therapy, *n* (%)	
Pyridostigmine	230 (92%)
Dose, mg, median (IQR)	240 (180–240)
Steroid	175 (70%)
Dose, mg, median (IQR)	25 (13–38)
Azathioprine	34 (13%)
Dose, mg, median (IQR)	100 (100–112.5)
Cyclosporine	5 (2%)
Dose, mg, median (IQR)	200 (200–225)

Abbreviations: AchR, acetylcholine receptors; IQR, interquartile range; MGFA, Myasthenia Gravis Foundation of America.

In addition to their standard MG therapy, 12 and 26 patients underwent plasmapheresis or intravenous immunoglobulins administration before operation, respectively.

The operation was accomplished in the majority (99%) of patients from the left side, with a median operative time of 135 min (Table 2). An additional contralateral fourth camera port was used in four cases, due to abundant mediastinal fat that did not allow for a safe dissection along the right phrenic nerve. In another case, a cervicotomy was performed to complete the dissection of the thymic upper horns in the neck.

**TABLE 2:** Surgical, postoperative, and pathological outcomes.

Variable	Study population (*n* = 267)
Side of surgery, *n* (%)	
Left	265 (99%)
Right	2 (1%)
Operative time, minutes, median (IQR)	135 (110–168)
Conversion, *n* (%)	7 (2.6%)
Postoperative complication, *n* (%)	20 (7.5%)
Myasthenic crisis	7
Hemothorax	3
Chylothorax	2
Pleural effusion	3
Fever	2
Port site infection	1
Pneumothorax	1
In‐hospital death, *n* (%)	0 (0%)
Postoperative chest tube LOS, median (IQR)	2 (1–2)
Postoperative in hospital LOS, median (IQR)	4 (3–5)
Histology, *n* (%)	
Thymic Hyperplasia	169 (63.2%)
Atrophic Thymus	18 (6.7%)
Normal Thymus	13 (4.9)
Thymoma	67 (25.2%)
A	3 (4.5%)
AB	10 (14.9%)
B1	19 (28.4%)
B2	23 (34.3%)
B3	12 (17.9%)
Masaoka‐Koga stage, *n* (%)	
I	14 (21%)
II	46 (68.6%)
III	4 (6%)
IV	3 (4.4%)
Weight of specimen, gr, median (IQR)	41 (28–61)
Ectopic thymus, *n* (%)	47 (18%)

Abbreviations: IQR, interquartile range; LOS, length of stay.

There were 7 (2.6%) conversions to open, in five cases because of a thymic tumor with suspicious infiltration either of the pericardium, the phrenic nerve, or of the vessels, in the other two cases because of technical difficulties that made robotic dissection unsafe. No emergency conversion was reported.

In the postoperative period, 20 (7.5%) patients experienced some type of surgical complication. Particularly, 7 (2.6%) patients had a postoperative myasthenic crisis, in one case leading to intubation and mechanical ventilation. Three patients had hemothorax but were all managed conservatively, without the need for reoperation. In one patient with persistent bilateral chylothorax, surgical duct ligation was necessary.

Pathological examination revealed follicular hyperplasia in 169 (63.2%) patients and thymoma in 67 (25.2%) cases (Table 2); the presence of thymic tumor was more frequent in seropositive patients (29% vs. 9.5%, *p* = 0.002) (Table S1). The median weight of the specimen was 41 g and, notably, there were no significant variations in the weight of the resected specimens (AME 0.20, 95% CI −0.55 to 0.97, *p* = 0.6) during the study period.

After a median follow‐up time of 83 months (IQR 31–143), 8 patients were dead (4 for extra‐thymic tumors, 2 for Covid‐19‐related complications and 2 for unknown causes), and 21 patients were lost to follow‐up. In the population with thymic tumors, there was one reported recurrence after 47 months in a patient with a radically resected Masaoka stage IV tumor.

**FIGURE 1:**
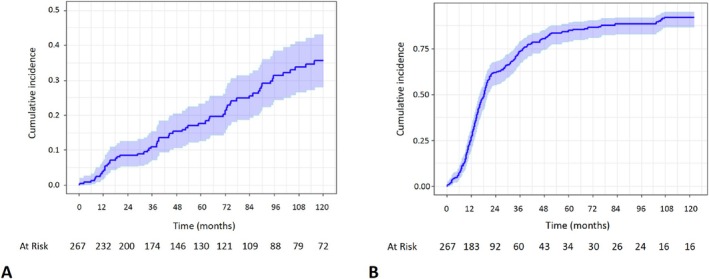
Cumulative incidence function curves of estimated complete stable remission (A) and Improvement (B) after thymectomy. The shaded area is a 95% confidence interval.

Among patients with available follow‐up, all were neurologically evaluated after surgery. According to the latest evaluation, 69 (25.8%) patients had a CSR, 23 (8.6%) had PR, and 125 (46.8%) were reported having only MM, thus with a general improvement rate of 81%. Estimated CSR and improvement incidence at 5 and 10 years after thymectomy were 18% and 36%, and 84% and 92%, respectively (Figure 1).

**FIGURE 2:**
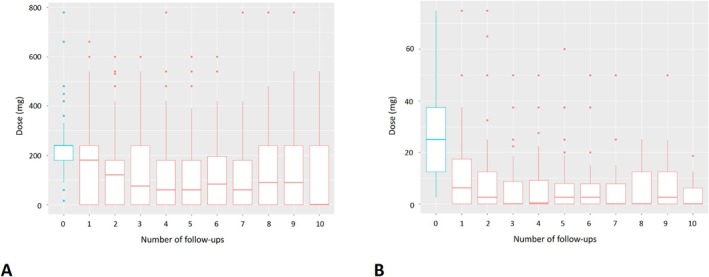
Postoperative reduction over time of the median dose of pyridostigmine (A) and steroids (B) compared with the basal preoperative treatment.

Among patients on preoperative pyridostigmine, 27% of them managed to reduce and 40% to interrupt the drug, and so did 36% and 42% of patients taking steroids, respectively. Significant reduction in the median pyridostigmine dose (AME −3.48, 95% CI −4.11 to −2.85, *p* < 0.001) and steroid dose (AME −1.27, 95% CI −1.70 to −0.84, *p* < 0.001) was observed over time (Figure 2).

**FIGURE 3:**
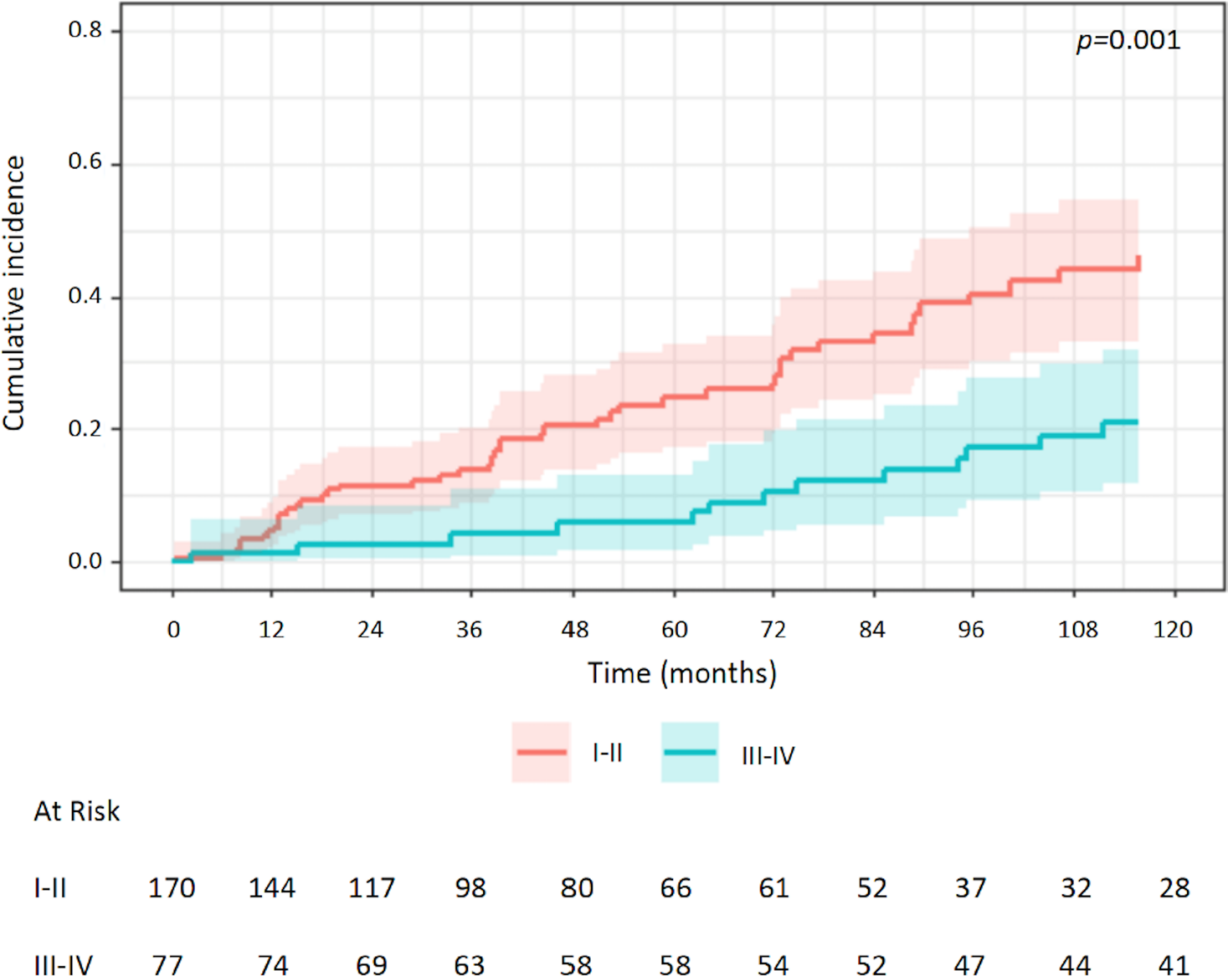
Cumulative incidence function curves of estimated complete stable remission after thymectomy stratified for Myasthenia Gravis Foundation of America class I‐II versus III‐IV. The shaded area is a 95% confidence interval.

Univariable analysis of prognostic factors for CSR showed a negative association with MGFA class III‐IV (vs. I‐II: HR 0.39, 95% CI 0.22–0.68, *p* = 0.001) (Figure 3), age (HR 0.98, 95% CI 0.96–1.00, *p* = 0.03), preoperative use of pyridostigmine (HR 0.24, 95% CI 0.10–0.54, *p* < 0.001) and of azathioprine (HR 0.42, 95% CI 0.19–0.93, *p* = 0.03). Instead, the year of surgery was positively associated with CSR (HR 1.13, 95% CI 1.07–1.19, *p* < 0.001). The effect of the MGFA class was significant also when considering MGFA class II‐III‐IV versus class I (HR 0.48, 95% CI 0.28–0.83, *p* = 0.008) (Figure S2). Multivariable analysis confirmed the association with year of surgery, use of pyridostigmine and age (Table 3). When multivariable analysis was performed without including the year of surgery as a variable, also MGFA class resulted significantly associated with CSR (III‐IV vs. I‐II: HR 0.41, 95% CI 0.23–0.73, *p* = 0.002) (Table S2).

**TABLE 3:** Univariable and multivariable logistic regression analysis of predictors for complete stable remission.

Characteristic	*N*	Univariable		Multivariable	
		HR (95% CI)	*p*	HR (95% CI)	*p*
Sex	265		0.215	—	—
F		—			
M		0.7 (0.39–1.23)			
Age	265	0.9 (0.96–1.00)	0.034	0.97 (0.95–0.99)	0.001
Year of surgery	265	1.13 (1.07–1.19)	< 0.001	1.11 (1.04–1.18)	0.001
MGFA	245		0.001		0.052
I‐II		—		—	
III‐IV		0.39 (0.22–0.68)		0.55 (0.3–1.01)	
Ab anti‐AchR	265		0.528	—	—
Positive		—			
Negative		1.18 (0.70–2.00)			
Symptoms duration	219	0.99 (0.98–1.00)	0.232	—	—
Pyridostigmine preoperative	249		< 0.001		0.014
No		—		—	
Yes		0.24 (0.10–0.54)		0.34 (0.15–0.81)	
Steroids preoperative	249		0.144	—	—
No		—			
Yes		1.44 (0.88–2.35)			
Azathioprine preoperative	249		0.032		0.258
No		—		—	
Yes		0.4 (0.19–0.93)		0.6 (0.28–1.41)	
Histology	265			—	—
Hyperplasia		—			
Thymoma		0.82 (0.42–1.58)	0.550		
Atrophic		0.48 (0.15–1.55)	0.221		
Normal		0.47 (0.12–1.94)	0.298		
Weight of specimen	200	1.00 (0.99–1.01)	0.779	—	—
Ectopic Thymus	257		0.479	—	—
No		—			
Yes		0.81 (0.45–1.46)			

Abbreviations: AchR, acetylcholine receptors; CI=confidence interval; HR, hazard ratio; MGFA, Myasthenia Gravis Foundation of America.

## 4 Discussion

The MGTX trial finally demonstrated the benefits of thymectomy compared with medical therapy alone in the treatment of MG. However, the results of the study are applicable only to patients with MGFA class II‐IV, positive AbAchR, non‐thymomatous MG, and undergoing transsternal thymectomy [4]. In the last decades, minimally invasive techniques have emerged as valuable alternatives to the transsternal approach, with a reduction of operative complications and apparently comparable neurological outcomes [5]. Among these new approaches, robotic surgery seems the most effective when dealing with a narrow and difficult‐to‐reach space like the mediastinum [7].

However, the role of minimally invasive thymectomy and the superiority of a technique over the others remain controversial. Heterogeneity in patients' selection, different timing and extent of surgery, and the different clinical classifications used have made comparisons impossible. Moreover, available reports are frequently characterized by small sample size and short duration of follow‐up [6, 7, 8, 9, 10, 11, 12].

The present analysis has the indubitable advantage of a large sample size, long follow‐up, and consistency through the years in terms of indications, technique, and extent of resection. In all cases, an extended thymectomy was performed. Our preference is for a left‐sided approach because of technical, clinical, and anatomical reasons [6, 16, 17]. Furthermore, in a recent publication, it was demonstrated that the left side approach seems favorably associated with a good neurological outcome [18]. We applied a right‐side approach only in two cases because of predominantly right‐sided thymomas. Moreover, as we described, a fourth port can be added if a better visualization of the right mediastinum is needed.

An indubitable advantage of the robotic system is represented by the enhanced visualization and wide range of movement of the robotic arms that allow for a precise and safe dissection. This is demonstrated by the low rate of open conversion (2.6%), comparable with previous reports (0%–2.9%) [6, 7, 9, 10, 11, 12]. It must be considered that these conversions were never performed in an emergency condition, but in all cases for which a thoracoscopic resection was deemed unsafe.

Regarding the neurological outcomes, the goal for the treatment of MG is to obtain a MM post‐intervention status or better [19]. Therefore, we considered all patients with MM, PR, or CSR in the improved MGFA‐PIS category and described an improvement rate of 84% after 5 years from the operation.

We also report a CSR rate of 18% at 5 years and 36% at 10 years, thus demonstrating an increased rate of remission over time. These data differ from the crude rate of remission (25.8%), revealing how the latter may be heavily biased by the length of follow‐up. As previously pointed out, time‐to‐event analyses seem the most reliable methods to evaluate improvement and remission incidence [3, 20]. Unfortunately, they are not used in all reports, thus it is impossible to make a definitive comparison [20]. Other confounding factors that may affect results and comparison between studies are the significant variations in severity and duration of preoperative illness, subgroups of analyzed population, neurological therapy and evaluation, and definitions of outcomes.

Another advantage of our study is the use of the established MGFA definitions as the standard reporting method. However, MGFA post‐intervention status is strictly dependent on the neurological evaluation, and there may be some subjectivity in the discrimination between classes II versus III (mild vs. moderate weakness) and III‐IV (moderate vs. severe weakness) that may have led to some bias because of potential differences between patients who had their neurological follow‐up inside vs. outside our Center. The implementation of more detailed outcome measures such as Quantitative Myasthenia Gravis score (QMG) or MG Activities of Daily Living (MG‐ADL) may help reduce bias; however, these outcomes are not less operator‐dependent.

Analyzing the risk factors for CSR, interestingly, we found that there is a significant influence of the year of surgery on the outcome, with patients operated on in the later part of our experience having a higher chance of remission. Although there haven't been exceptional changes in the field of myasthenia treatment during these last 20 years, a possible explanation could be the so‐called “cohort effect” This takes into account different marginal gains that may have been introduced during the long study period as, for example, the increased surgical experience, the improved robotic systems, and the better care of these patients by the neurologists.

This finding has also a surprising effect on the relationship between MGFA class and CSR. Indeed, if we do not consider the year of surgery in the model, we find that advanced MGFA class (III‐IV) patients have a worse prognosis in terms of remission (Table S2), consistent with previous reports [18, 21, 22]. On the contrary, if we introduce the era of surgery, MGFA class is no more statistically significant. Probably, the better care that we are now able to give to these patients overcomes the negative impact of severe MGFA class.

On the other side, controversy persists regarding the indication for thymectomy in patients with MGFA class I, although about 60% of them will ultimately progress to a generalization of symptoms and the significant side effects of the immunosuppressive treatments. Moreover, Li and colleagues have demonstrated that the rate of CSR in MGFA I patients is higher if operated on before generalization [8, 23]. Unfortunately, we can't support this finding as we don't actually know how many patients had only ocular symptoms at the beginning of the disease and eventually progressed to a generalized form, as we recorded only the preoperative MGFA. Among MGFA class I patients, we recorded just 2 (3.5%) cases that had a worsening of symptoms after surgery, one with generalization of MG. Because of these considerations, we offer thymectomy also to MGFA class I patients after thorough discussion with the patient and the referring neurologist. Furthermore, among these patients we reported only two complications (3.5%) and a CSR incidence at 5 years of 28% and at 10 years of 63% (Figure S2), therefore we are confident that we are offering them a safe and effective operation.

In our series, we failed to demonstrate the association between CSR and histology, seropositive MG, early operation after symptoms onset, weight of the specimen, and presence of ectopic thymic tissue. Regarding these two last points, we can make some considerations. First of all, the meaning of the finding of ectopic thymic tissue and of its removal is still ambiguous. According to different studies, its presence seems to negatively relate to symptoms' remission, while others did not find these results [22, 24]. Also, in our series, we did not find an association between the presence of ectopic tissue and the neurological outcomes. We must consider that the presence of ectopic foci is quite low in our study (18%), although this could also be related to the low number of patients with normal or atrophic thymus (in whom the ectopic foci seem to be more frequent).

Also the weight of the specimen has been proposed as a surrogate of the extent of mediastinal resection and as a predictor of CSR [25]. In our series, the median weight was 41 g, which is comparable with other series [25] whereas Rückert, for example, found a significantly heavier specimen's weight [12, 17, 26]. It must be considered that we did not correct the weight for the body surface area, and this could explain the differences in weight that can be found when dealing with different populations/ethnicities. Additionally, we found that the weight of the specimen was substantially stable during our study, demonstrating that the extent of the resection has been consistent over the years.

Surprisingly, we found that preoperative pyridostigmine use affects the rate of CSR. Acetylcholinesterase inhibitors are the first‐line drugs in the management of MG, but represent only a symptomatic treatment [19]. We speculate that patients whose preoperative therapy did not include pyridostigmine could have a distinct and milder form of MG, with optimal response to thymectomy. As a matter of fact, 19/21 patients with no use of pyridostigmine were classified as MGFA I or II. However, it must be also considered that, as previously mentioned, the preoperative treatment is based on the subjective evaluation of the single neurologist. Furthermore, because of the small number of patients not treated with pyridostigmine, this could represent a false positive result.

In contrast to the MGTX, our case series also includes seronegative patients, who clearly benefited from thymectomy, reinforcing the concept that seronegative patients should be considered for thymectomy, especially if they present generalized/bulbar weakness and early onset. Our data are consistent with previous studies that found no difference in neurological outcomes between seropositive and seronegative patients [27, 28]. We also described some differences in the two groups of patients, with seronegative ones being younger and, not surprisingly, with a lower incidence of thymoma. Instead, disease severity was similar, although there were slightly more patients with MGFA class IV disease who were treated with cyclosporine.

Furthermore, the concept of seronegative MG is itself debated. Indeed, we routinely test only AbAchR and, if negative, also anti‐MuSK. If both are negative, the patient is considered seronegative. However, the implementation of new and more sensitive assays compared with the RIPA (radioimmunoprecipitation assay) test that is used in our center, such as fixed and live cell‐based assays (CBA), may further reduce the proportion of true seronegativity in patients with MG in the future. Additionally, it must be considered that other antibodies, not routinely tested in clinical practice, can be found in patients with MG (anti‐LRP4, anti‐Kv1.4, anti‐RyR, anti‐titin), although their role in the pathogenesis of the disease and their impact on the response to thymectomy is not clear [22].

Therefore, among the so‐called seronegative patients, there could be one or more subpopulations of patients that are false seronegative or express other antibodies, and could differently respond to surgery, explaining the different results in the literature.

This work has some limitations, mainly based on the retrospective nature of the study, which is subject to biases inherent to its design. Although the data were prospectively collected on consecutive patients, it would be impossible to identify all the confounders in a retrospective fashion. Neurological outcomes may also be influenced by the different therapeutic regimens applied in the preoperative and postoperative settings. On the other hand, the consistency of the surgical technique over time in a high‐volume center and performed by the same group of surgeons, the large sample size, and the long follow‐up time are indubitable advantages of this work.

In conclusion, this large 20 years' single‐center experience showed that extended robotic thymectomy in patients with MG is a safe surgical procedure. Long‐term neurological follow‐up shows high efficacy, with remission or reduction in medication in most patients, also in those subgroups that historically showed worse outcomes, as patients with thymoma, seronegative disease or advanced MGFA class. A prospective evaluation with a standardized surgical procedure and neurological evaluation, and outcomes' analysis, possibly including MGFA class 1 and seronegative patients, is still necessary to define the role of minimally invasive thymectomy in myasthenia gravis.

## Author Contributions

**Giovanni M. Comacchio:** conceptualization, methodology, data curation, investigation, writing – original draft, writing – review and editing, resources, project administration. **Marco Schiavon:** conceptualization, investigation, writing – original draft, methodology, writing – review and editing, project administration, data curation, resources. **Luca Bello:** conceptualization, investigation, writing – review and editing, methodology, data curation. **Marco Mammana:** conceptualization, investigation, methodology, writing – review and editing, validation, data curation. **Eleonora Faccioli:** conceptualization, investigation, methodology, data curation, writing – review and editing. **Elena Pegoraro:** conceptualization, investigation, methodology, data curation, supervision, writing – review and editing. **Giulia Lorenzoni:** methodology, software, formal analysis, validation, visualization, writing – review and editing. **Giorgio Cannone:** data curation, writing – review and editing, investigation. **Giuseppe Cataldi:** writing – original draft, investigation, data curation, resources. **Giulia Pagliarini:** investigation, data curation, writing – original draft, resources. **Alessandro Rebusso:** data curation, investigation, writing – review and editing. **Samuele Nicotra:** investigation, data curation, writing – review and editing. **Dario Gregori:** formal analysis, writing – review and editing, validation, software. **Maria Carlotta Marino:** data curation, investigation, writing – original draft, writing – review and editing, resources. **Giuliana Capece:** data curation, investigation, writing – review and editing, resources. **Pietro Riguzzi:** data curation, investigation, writing – review and editing, resources. **Federica Pezzuto:** data curation, investigation, writing – review and editing, resources. **Fiorella Calabrese:** conceptualization, supervision, data curation, writing – review and editing, investigation. **Andrea Dell'Amore:** conceptualization, investigation, writing – review and editing, data curation, supervision. **Federico Rea:** supervision, data curation, investigation, conceptualization, methodology, writing – review and editing, project administration.

## Conflicts of Interest

The authors declare no conflicts of interest.

## Supporting information


**Figure S1:** Number of patients operated on during each of our 20 years’ activity (April 2002–April 2022) and subdivided among thymomatous and non‐thymomatous.

**Figure S2:** Cumulative incidence function curves of estimated Complete Stable Remission after thymectomy stratified for Myasthenia Gravis Foundation of America class I versus II‐III‐IV. The shaded area is a 95% confidence interval.

**Table S1:** Characteristics of seropositive and seronegative patients.

**Table S2:** Multivariable logistic regression analysis of predictors for Complete Stable Remission, excluding the year of surgery from the model.

## Data Availability

The data that support the findings of this study are available on request from the corresponding author. The data are not publicly available due to privacy or ethical restrictions.
