# Supplementary figures and images for: Robotic Thymectomy for Myasthenia Gravis: Analysis of the Surgical and Neurological Outcomes After a 20 Years' Experience
Source: Eur J Neurol. 2025 Apr 15;32(4):e70147. doi: 10.1111/ene.70147 (PMC11998024; doi:10.1111/ene.70147)

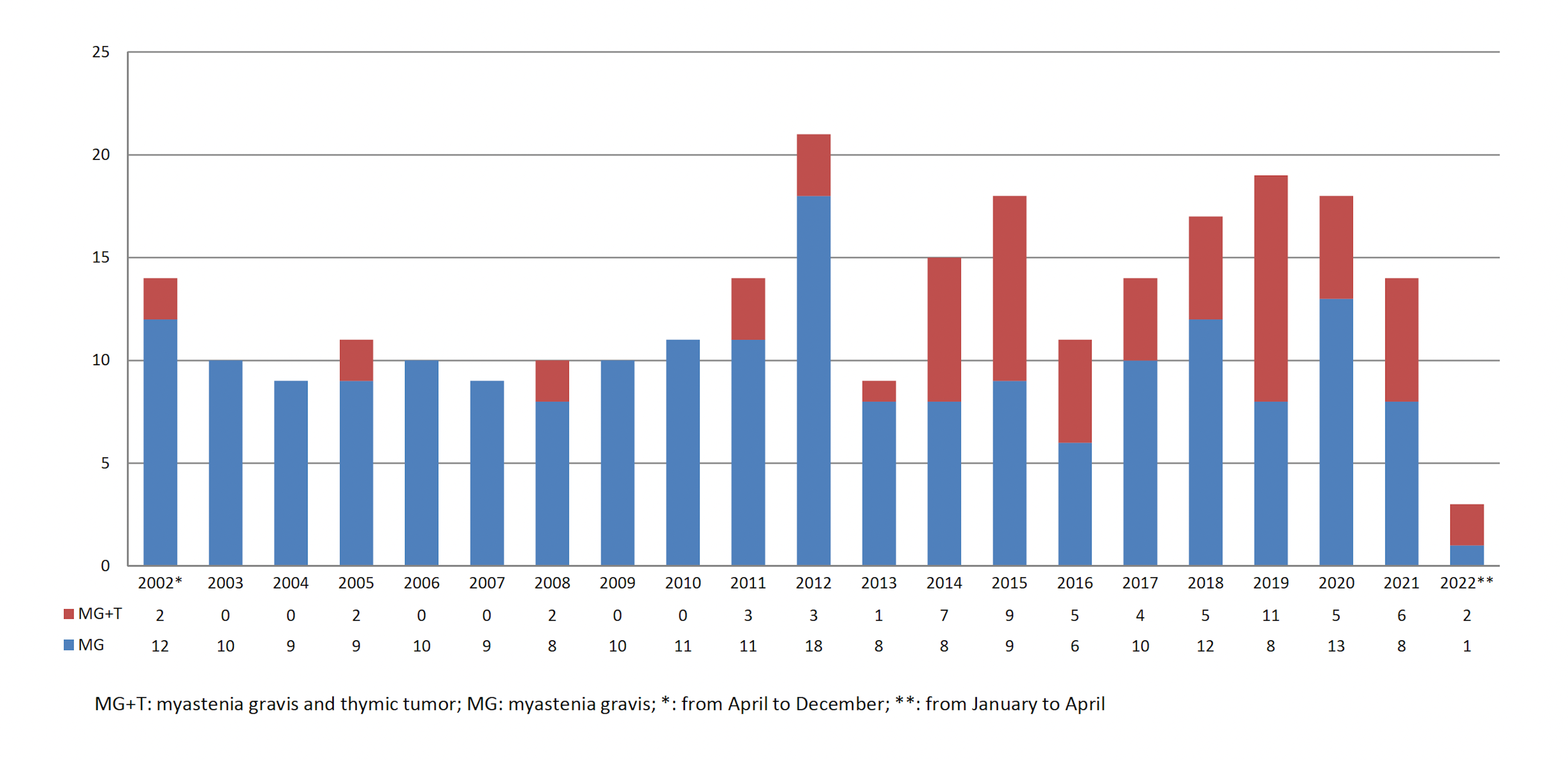

Supplement: Supplementary file 1 — Figure S1: Number of patients operated on during each of our 20 years’ activity (April 2002–April 2022) and subdivided among thymomatous and non‐thymomatous. [file ENE-32-e70147-s004.tif]
