# Supplementary material for: Robotic Thymectomy for Myasthenia Gravis: Analysis of the Surgical and Neurological Outcomes After a 20 Years' Experience
Source: Eur J Neurol. 2025 Apr 15;32(4):e70147. doi: 10.1111/ene.70147 (PMC11998024; doi:10.1111/ene.70147)
Supplement: Supplementary file 3 — Table S1: Characteristics of seropositive and seronegative patients. [file ENE-32-e70147-s001.doc]

**Supplementary Table 1:** Characteristics of seropositive and seronegative patients

| **Variable** | **Seropositive**  **(n=198)** | **Seronegative**  **(n=63)** | **p-value** |
| --- | --- | --- | --- |
| **Sex, n(%)**  ***F***  ***M*** | 135(68%)  63(32%) | 46(73%)  17(27%) | 0.5 |
| **Age at surgery, years, median(IQR)** | 44(33-56) | 36(26-48) | 0.003 |
| **Preoperative MGFA, n(%)**  ***I***  ***II***  ***III***  ***IV*** | 37(21%)  88(49%)  45(25%)  9(5%) | 15(24%)  25(40%)  14(22%)  9(14%) | 0.1 |
| **Symptoms duration, months, median(IQR)** | 11(6-20) | 13(7-22) | 0.4 |
| **Preoperative therapy, n(%)**  ***Pyridostigmine***  ***Steroid***  ***Azathioprine***  ***Cyclosporine*** | 169(92%)  133(72%)  26(14%)  1(0.5%) | 56(90%)  39(63%)  11(18%)  4(6.5%) | 0.7  0.2  0.5  0.01 |
| **Histology, n(%)**  ***Thymic Hyperplasia***  ***Atrophic Thymus***  ***Normal Thymus***  ***Thymoma*** | 115(58%)  14(7%)  12(6%)  57(29%) | 52(83%)  4(6%)  1(1.5%)  6(9.5%) | 0.002 |
| **Weight of specimen, gr, median(IQR)** | 41(28-64) | 41(30-52) | 0.8 |
| **Ectopic thymus, n(%)** | 32(17%) | 12(19%) | 0.7 |

AchR=acetylcholine receptors; IQR=Interquartile range; MGFA=Myasthenia Gravis Foundation of America.
