# Supplementary material for: Robotic Thymectomy for Myasthenia Gravis: Analysis of the Surgical and Neurological Outcomes After a 20 Years' Experience
Source: Eur J Neurol. 2025 Apr 15;32(4):e70147. doi: 10.1111/ene.70147 (PMC11998024; doi:10.1111/ene.70147)
Supplement: Supplementary file 4 — Table S2: Multivariable logistic regression analysis of predictors for Complete Stable Remission, excluding the year of surgery from the model. [file ENE-32-e70147-s003.doc]

**Supplementary Table 2:** Multivariable logistic regression analysis of predictors for Complete Stable Remission, excluding the year of surgery from the model

| **Characteristic** | **N** | **HR (95%CI)** | ***p*-value** |
| --- | --- | --- | --- |
| **Age** | 265 | 0.98(0.96-1.00) | 0.014 |
| **MGFA** | 245 |  | 0.002 |
| ***I-II*** |  | — |  |
| ***III-IV*** |  | 0.41(0.23-0.73) |  |
| **Pyridostigmine preoperative** | 249 |  | 0.001 |
| ***No*** |  | — |  |
| ***Yes*** |  | 0.24(0.10-0.56) |  |
| **Azathioprine preoperative** | 249 |  | 0.092 |
| ***No*** |  | — |  |
| ***Yes*** |  | 0.5(0.23-1.12) |  |

MGFA=Myasthenia Gravis Foundation of America;HR=Hazard Ratio;CI=Confidence Interval.
